# Influence of temperature on intraspecific, unbalanced dyadic contests between crabs

**DOI:** 10.7717/peerj.7845

**Published:** 2019-10-23

**Authors:** Allan T. Souza, Felipe O. Ribas, João F. Moura, Claudia Moreira, Joana Campos, Martina I. Ilarri

**Affiliations:** 1 Biology Centre of the Czech Academy of Sciences, v.v.i., Institute of Hydrobiology, České Budějovice, Czech Republic; 2 CIIMAR—Interdisciplinary Centre of Marine and Environmental Research, University of Porto, Novo Edifício do Terminal de Cruzeiros do Porto de Leixões, Matosinhos, Portugal; 3 ICBAS/UP—Abel Salazar Biomedical Sciences Institute, University of Porto, Porto, Portugal

**Keywords:** Climate change, Morphology, Ecology, Shore crab, Aggressive behavior

## Abstract

Intraspecific agonistic interactions are widespread across the animal kingdom, with many individual morphological and physiological characteristics playing important roles in the fate of disputes. Additionally, changes to environmental conditions can influence the outcomes of animal contests. The shore crab (*Carcinus maenas*) is a globally distributed species, present in numerous coastal and estuarine temperate systems around the world. Although shore crabs are highly tolerant to changes in temperature, this parameter has important physiological effects on the species’ ecology, while its effects on behavior are not fully understood. Our study aims to investigate how different individual characteristics (such as sex, color morphotype, carapace and chela morphology) and temperature conditions affect the dyadic interactions between shore crabs when disputing food resources. In general, the differences in carapace width between opponents, their sexes, color morphotypes and the temperature conditions interacted and were important predictors of the contest fate. We found that the body size and color morphotype of *C. maenas* determined the fate of dyadic disputes. However, the higher temperatures disrupted the well-established dominance of the larger red color morphotype individuals. Overall, the agonistic contest results suggest higher plasticity than previously acknowledged.

## Introduction

Animal conflicts are common across many taxa (e.g.,  Bonacci et al., 2006; Ferry et al., 2016; Robins, 2008; Robinson, 2014; Souza, Ilarri & Rosa, 2011; Tierney, Godleski & Massanari, 2000) and can be either inter or intraspecifically orientated. Bouts over food resources and territorial disputes are common inter and intraspecific interactions (e.g.,  Dröscher & Kappeler, 2014; Souza, Ilarri & Rosa, 2011), while disputes for mates, protection of offspring and social hierarchy inside a given population are exclusive intraspecific interactions (e.g.,  Lee, Yang & Curley, 2018; Sato & Nagayama, 2012; Souza & Ilarri, 2014).

Another important difference between inter and intraspecific interactions is the difference in size between contestants of different species. Contestants in intraspecific conflicts are more equally matched although variability still exists between life stages (e.g.,  Baeza, Stotz & Thiel, 2002; Moore et al., 2014), sexes (e.g.,  Warren et al., 2009) or physiological states (e.g.,  Barki, Karplus & Goren, 1991; Ventura, Rosen & Sagi, 2011).

Resource holding potential (RHP) is a theoretical term that explains the ability of an individual to hold or gain a contested resource and to avoid or to inflict contest costs dependent on a set of individual characteristics. Asymmetries among individuals create different levels of RHP that will eventually alter the fate of pairwise conflicts. Across taxa, different features, such as body size and weight, bite or pinching strength, size, shape and robustness of special body parts used in conflicts, behavioral tactics and experience, create advantages for individuals in conflicts (Ayres-Peres, Araujo & Santos, 2011; Bonacci et al., 2006; Briffa & Sneddon, 2007; Husak et al., 2006; Lailvaux et al., 2005; Sneddon et al., 2000; Souza et al., 2011). Additionally, animals can differ in their tolerance levels to even minor changes in the environment, which can influence their competitive interactions with other individuals (Gherardi et al., 2013).

Previous studies suggest that changes in climatic conditions can vary the strength of behavioral interactions between individuals (Gherardi et al., 2013). Temperature, in particular, has the potential to influence the behavior of many species across diverse taxa, since it directly affects metabolism, feeding ability and growth rates of ectotherms (Hartnoll, 2001; Petry et al., 2007).

The shore crab (*Carcinus maenas*) is a globally distributed species, present in numerous coastal and estuarine temperate systems around the world (Roman & Palumbi, 2004). Despite being highly tolerant to temperature changes (Cohen, Carlton & Fountain, 1995; Klassen & Locke, 2007), they can be affected by temperature variations that change their ecological roles (e.g.,  McGaw & Whiteley, 2012; Taylor, Butler & Al-Wassia, 1977; Welch, 1968) resulting in the failure to establish viable populations in warm waters around the world (see Klassen & Locke, 2007). Sneddon, Huntingford & Taylor (1997a) demonstrated that the magnitude and direction of intraspecific, agonistic interactions of shore crabs is dependent on the population density, and affects the competition for food, mates and shelter. Due to the high population densities in which shore crabs are often found (Baeta et al., 2006; Raffaelli et al., 1989; Souza, 2013), aggressive interactions are common to settle intraspecific conflicts. Dissimilarities in agonistic behaviors between crustaceans can result in uneven access to resources, in which the most aggressive individuals have an advantage (Briffa, 2013). The present study aims at investigating how different individual characteristics (such as sex, color morphotype, carapace and chela morphology) and temperature conditions affect the dyadic interactions among shore crabs when disputing food resources.

## Materials & Methods

### Maintenance of study animals

Shore crabs were collected in the Minho estuary (NW Iberian Peninsula) using a beam trawl and then transferred to the CIIMAR laboratory in Porto (Portugal). The crabs were kept in quarantine for approximately four weeks to ensure that all individuals were healthy and in good condition for the ethological experiment. During this time they remained in a holding tank supplied with recirculating brackish water (salinity ranging between 25 and 30 psu) kept at a constant temperature of 16 °C. After the quarantine ended, the experiment began and three different types of tanks were used (the summary of the maintenance conditions is presented in Table 1).

**Table 1:** Summary of the maintenance conditions of the shore crabs *Carcinus maenas* used on the experimental study.

**Tank characteristics**	**Holding tank**	**Isolation tank**	**Observation tank**
Capacity (Crabs)	50	1	2
Feeding regime	Twice per week	No feeding	Food dispute
Food per feeding event	50 g	–	3 g
Permanence period	7 days	7 days	5 min
Photoperiod (Day:Night)	12 h:12 h	12 h:12 h	12 h:12 h
Oxygenation	Constant	Constant	Constant
Water circulation and filtration	Constant	Daily	Constant
Salinity (psu)	25–30	25–30	25–30
Substrate	Sand	Gravel	Gravel
Temperature	16 °C	Variable^*^	Variable^*^
Volume (L)	320	2	45

**Notes.**

* The temperature remained fixed for one week in the isolation and the observation tanks at one of the five temperature levels (10, 13, 16, 19 and 22 °C).

**Figure 1:**
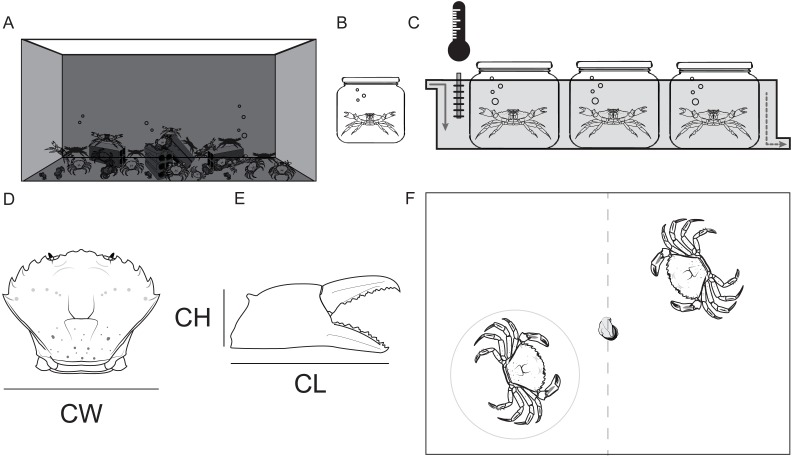
Maintenance of study animals. (A) Holding tank (320L). (B) Isolation tank (2L). (C) Observation tank with constant temperature and recirculating sea water. (D) Shore crab *Carcinus maenas* measurements: Carapace width (CW), (E) Chela height (CH) and Chela length (CL). (F) Arena for the contest for food (a piece of defrosted mussel); observed crab in the gray circle.

Crabs in the holding tank were fed with mussels twice a week (Fig. 1A). Once a week, 32 crabs were haphazardly selected and transferred to the enumerated isolation tanks (2 L) (Fig. 1B).

The selected crabs were kept in isolated conditions (physically, chemically and visually) for 7 consecutive days prior to any behavioral observations. The isolation tanks were placed partially submersed in recirculating sea water in a larger tank. This larger tank also had a heating/cooling system that kept the temperature constant at the target temperature levels (10, 13, 16, 19 and 22 °C), starting with 10 °C and ending at 22 °C. Crabs were starved during the isolation period. We selected this 7 day regime since *C. maenas* can be deprived of food for up to a 3 month period, so 7 days without nourishment would keep the crabs healthy and motivated to fight over food (Wallace, 1973). Only intact crabs (with hard exoskeleton, no epifaunal growth and no missing or recently regenerated limbs) were selected for the behavioral observations.

Before each contest, the carapace width of the crabs (between the fourth and fifth lateral spines) was measured to the nearest 0.1 mm, using vernier calipers. This was repeated also for the chela length and height of both claws (Fig. 1C). Afterwards, each chela was classified as the master (the one with larger propus length) or secondary chela (Elner, 1978).

Permission to collect shore crabs in the Minho estuary (NW Iberian Peninsula) was granted in correspondence with a captain of the Portuguese Navy.

### Experimental protocol

Crabs from different sex and color morphotypes (green and red) were studied (Females: Green = 14, Red = 2; Males: Green = 49, Red = 19). Two crabs were haphazardly selected from the isolation tanks and placed inside the observation tank. Prior to each contest each crab had 5 min to settle and acclimatize to the new tank. During this period the crabs were kept separated by an opaque divider in the middle of the tank to avoid visual contact before the contests (Fig. 1D). After the acclimatization period, the divider was removed and an individual whole mussel (food resource) was placed in the center of the tank in order to start the 5 min dyadic food contest. The duration of the contests was defined based on observations collected in the pilot study.

**Figure 2:**
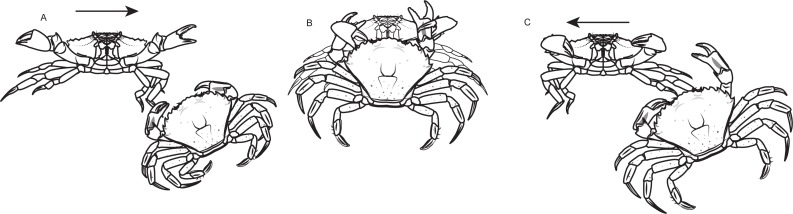
Standard agonistic behaviors of the shore crab *Carcinus maenas*. (A) Approach (when the crab moves towards the other crab without physical contact among them). (B) Attack (when the crabs have physical contact, and the observed individual hits out the other with one or both chelas, or use them to pinch the carapace, chelas or legs of the other crab). (C) Retreat (when the observed crab moves away from the opponent avoiding physical contact).

During the assays only one crab had its behavior visually recorded (hereafter referred as the observed crab). The contests were separated into three contexts: before (i.e period before one of the two crabs grabbed the food), with (i.e period when the observed crab kept the food) and without (i.e., period when the non-observed crab held the food). It was possible for the observed crab to switch between the “with” and “without” contexts during the dyadic contest. The duration of each context varied among contests, since they depended on the behavior of each pair of crabs in the contest. If one crab lost control over the food after an initial possession, and its opponent did not grab the food item within 3 s, the agonistic interactions in this instance would have been assigned a different contest context. However this situation never occurred. During each context three types of standard behaviors were considered: approach (i.e., when the crab moved towards the other crab without physical contact between them) (Fig. 2A), attack (i.e., when the crabs had physical contact, and the observed individual hit the other with one or both chelae, or used them to pinch the carapace, chelae or legs of the other crab) (Fig. 2B) or retreat (i.e., when the observed crab moved away from the opponent avoiding physical contact) (Fig. 2C). Each agonistic interaction was a unique event that was defined by an uninterrupted action, meaning that if the crab stopped its standardized movement and re-initiated it immediately after, then two events were recorded. Retreats were classified as defensive interactions, while approach and attack were grouped as aggressive interactions. Those behaviors were previously established and modified based on a pilot study and on the literature (Sneddon, Huntingford & Taylor, 1997a). If the food resource was divided among the crabs during the dyadic contest, this bout was discarded from the dataset. The crab who kept the resource at the end of the 5 min dyadic contest was considered the winner. All the recordings were performed by the same person (ATS) in order to avoid human observer bias. The observer was always located 1.5 m away from the observation tank with a front view. After the end of the experiments crabs were kept in the holding tank for two weeks to make sure that they were not in proecdysis.

### Data analysis

Generalized mixed effects models were used to describe the behavior of *C. maenas* during their intraspecific food disputes. The models were fitted using the *glmer* function from the *lme4* package (Bates et al., 2015). In order to avoid the inclusion of correlated variables into the model, the morphometric variables (carapace width, chela length and height) were tested for correlation (Pearson’s correlation) using the *findCorrelation* function in the *caret* package (Kuhn et al., 2018). Given that the morphometrical measurements were correlated, only the carapace width was selected to be included into the model because it better describes the size of the crabs. Due to the nature of the data, the dependent variable was translated into a binary code (crab winning the dispute = 1 and crab losing the dispute = 0). The model predictors accounted for the interaction between the number of agonistic interactions, their type (Approach, Attack and Retreat) and the context of the food dispute (Before,Without and With) when it occurred, plus the morphometric descriptor (carapace width difference among opponents in mm). The water temperature level at time of the food dispute, the information about sex (Female:Female, Female:Male, Male:Female or Male:Male) and color morphotype (Green:Green, Green:Red, Red:Green or Red:Red) of the contestants were included in the models as random factors to account for the behavioral differences associated to temperature, sex and color morphotypes.

Prior to the analysis the numerical variables were cubic transformed in order to improve the homogeneity of the data. Models were tested for overdispersion using the *dispersion_glmer* function on *blmeco* package (Korner-Nievergelt et al., 2015). All statistical analyses were performed in R software (R Core Team, 2018).

## Results

We observed 84 interactions, of which 51 (60.7%) involved only males (Male:Male), 30 (35.7%) were between sexes (Male:Female), and only 3 (3.6%) were between females (Female:Female). When analyzing color morphotypes, 47 disputes (56.0%) involved only green crabs (Green:Green), 32 (38.0%) involved green and red crabs (Green:Red), and 5 (6.0%) involved exclusively red morph crabs (Red:Red).

The average carapace width (CW) of the *C. maenas* studied was 43.64 ± 5.62 mm (mean ± SD), with values ranging from 31.62 to 61.30 mm. The majority of crabs (81%) had the master chela on the right arm. The average length of the master chela was 23.69 ± 4.01, with values ranging from 16.70 to 36.90 mm, whereas the average length of the secondary chela was 21.27 ± 4.55 mm, with the values ranging from 15.04 to 35.26 mm. The average height of the master chela was higher (9.37 ± 1.86 mm) than the secondary chela (8.32 ± 1.74 mm).

**Figure 3:**
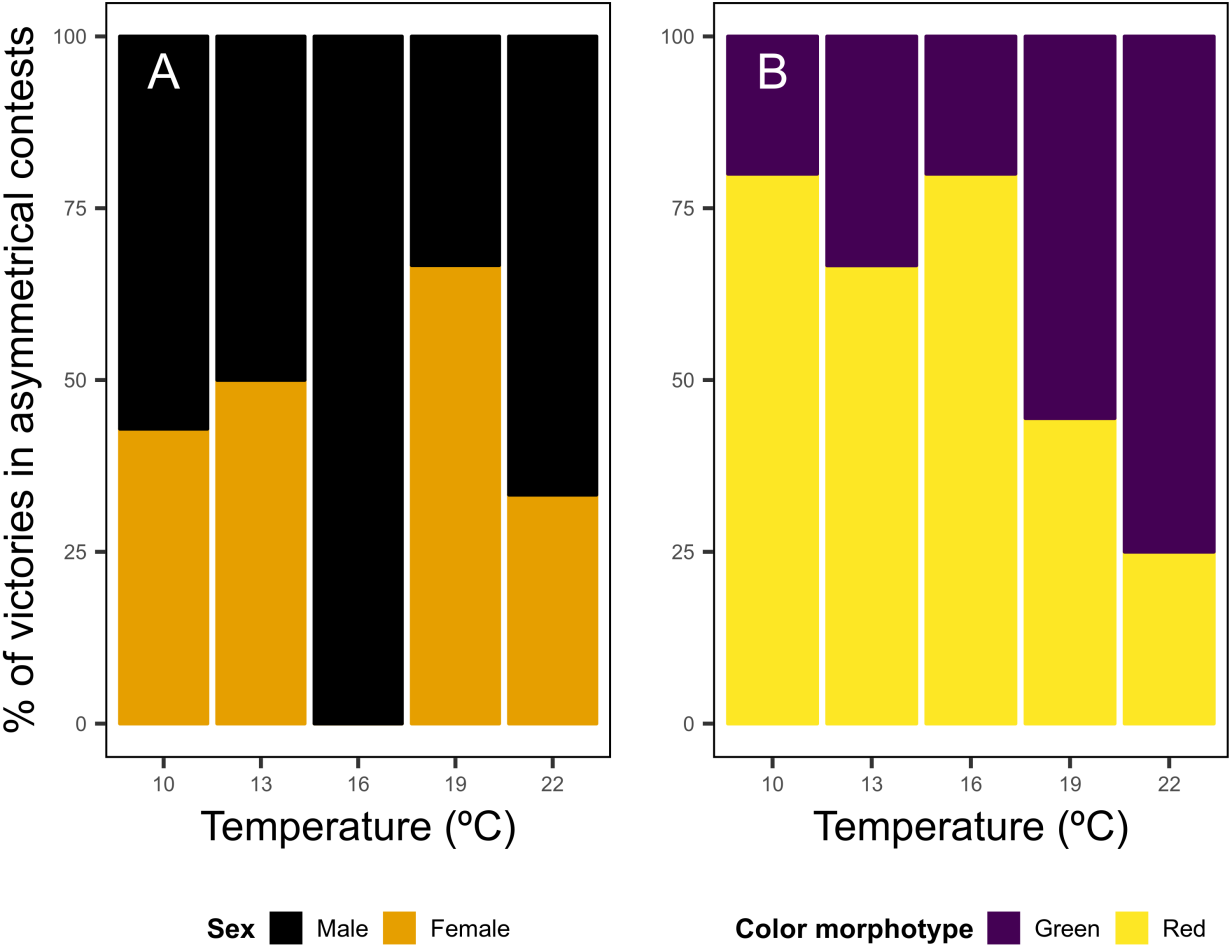
Asymmetrical dyadic contests involving shore crab (*Carcinus maenas*) individuals of different sexes (A) and color morphotypes (B) at different levels of water temperature.

In asymmetrical contests regarding the sex of individuals, males won 56.7% of the disputes, while females held the resource in 43.3% of the disputes, with no clear patterns across temperature levels noticed (Fig. 3A). Red shore crabs were victorious in 59.4% of asymmetrical bouts, and the red color morphotype was the most successful in contests for food. However the bout fate shifted according to water temperature, going from 80% of victories of the red morphotype in colder waters to 25% in warm waters (Fig. 3B).

**Figure 4:**
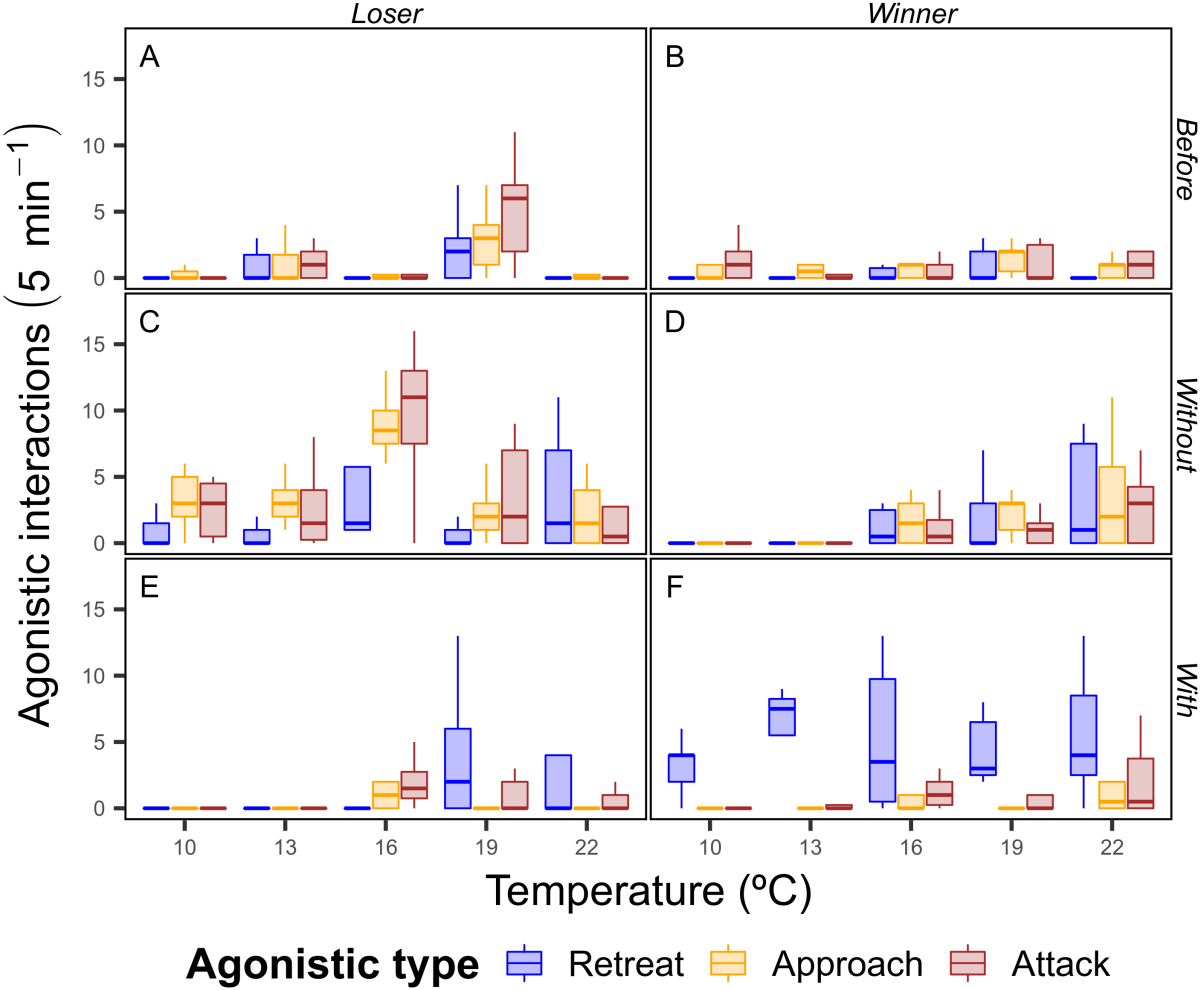
Total number of agonistic interactions displayed by the shore crab (*Carcinus maenas*) in disputes for food resource. Only one individual had their behavior recorded during the dispute, with the crab holding the food after the 5 minutes period being considered the winner (B, D and F) of the dispute (losers in A, C and E). Sub-panels also indicate the periods of the dispute (Before: A and B; Without: C and D; With: E and F—see ‘Material & Methods’ for definitions), whereas the colors indicate the type of agonistic interaction (see Fig. 2 for definitions).

There was a trend of increasing agonistic interactions as the temperature rose, with more interactions of all types for both winning and losing crabs at higher temperatures. Interestingly, at 10 and 13 °C the differences between the winners and losers of the food dispute were strong in the contexts with and without, where the winning crabs had a greater number of retreats with the food and the losing crabs had a higher number of interactions of all types than the winning crabs, which indicates that winning crabs were able to easily defend the food resource (Fig. 4).

The mixed-effects model showed that the carapace width was an important predictor for the food dispute outcomes, and that crabs that attacked more when they were in possession of the food were more often victorious. Overall color morphotype, temperature and sex were important drivers of crab behavior, accounting for 48% of the explanatory power of the model (Table 2).

**Table 2:** Summary of the mixed-effects logistic regression model applied to the dispute for food between two shore crab (*Carcinus maenas*) individuals.

	*Contest result*		
***Predictors***	***Odd Ratios***	***CI***	***p***
(Intercept)	1.37	0.24–7.95	0.727
Interactions	1.07	0.44–2.61	0.883
Approach	0.87	0.35–2.12	0.753
Attack	1.05	0.44–2.51	0.910
Without	1.18	0.50–2.78	0.708
With	0.23	0.08–0.66	**0.006**
Carapace difference (mm)	1.46	1.29–1.64	**<0.001**
Interactions*Approach	1.26	0.37–4.21	0.712
Interactions*Attack	0.89	0.29–2.77	0.840
Interactions*Without	0.74	0.25–2.22	0.589
Interactions*With	4.42	1.45–13.49	**0.009**
Approach*Without	3.50	0.87–14.00	0.077
Approach*With	5.55	1.41–21.90	**0.014**
Attack*Without	1.76	0.49–6.34	0.391
Attack*With	4.06	1.01–16.32	**0.048**
Interactions*Approach*Without	0.26	0.06–1.22	0.089
Interactions*Approach*With	0.12	0.02–0.65	**0.014**
Interactions*Attack*Without	0.53	0.12–2.27	0.394
Interactions*Attack*With	0.26	0.06–1.12	0.085


***Random Effects***	
*σ*^2^	3.29
*τ*_00temperature_	1.06
*τ*_00sex_	0.69
*τ*_00color_	1.23
ICC	0.48
Marginal/Conditional R^2^	0.126/0.541

**Notes.**

CI 0.95 confidence interval *σ*^2^ within group variation *τ*_00_ between group variation on intercept ICC interclass coefficient (proportion explained by the grouping structure)

Values in bold refer to *p* < 0.05.

When temperature data were split among cold waters (10 to 16 °C) and warm waters (19 to 22 °C) the mixed-effects model also revealed a different contribution of crab morphology into the food disputes. The model indicated that the crab morphology was not a good predictor of the outcome of the food disputes in warm waters and that intraspecific disputes between color morphotypes were highly variable, with the overall predictability of food disputes outcomes being more variable and not dependent on individual size, which differed from the pattern observed in colder waters (Table 3).

**Table 3:** Summary of the mixed-effects logistic regression model applied to the dispute for food between two shore crab (*Carcinus maenas*) individuals at two different levels of water temperature (cold = 10 to 16 °C, warm = 19 to 22 °C).

	*Cold waters*			*Warm waters*		
***Predictors***	***Odd Ratios***	***CI***	***p***	***Odd Ratios***	***CI***	***p***
(Intercept)	0.28	0.03–2.39	0.244	5.42	0.43–68.93	0.193
Interactions	1.48	0.48–5.09	0.529	0.32	0.08–1.22	0.096
Approach	0.76	0.23–2.43	0.639	0.76	0.18–3.18	0.704
Attack	0.97	0.31–2.97	0.951	1.05	0.27–4.15	0.941
Without	1.14	0.37–3.49	0.822	0.70	0.17–2.80	0.612
With	0.27	0.07–1.02	0.054	0.25	0.04–1.44	0.121
Carapace difference (mm)	1.69	1.44–2.00	**<0.001**	0.93	0.77–1.12	0.451
Interactions*Approach	1.49	0.28–8.03	0.645	2.55	0.43–15.15	0.303
Interactions*Attack	0.96	0.18–5.09	0.960	1.67	0.33–8.52	0.534
Interactions*Without	0.66	0.14–3.15	0.599	2.90	0.57–14.81	0.200
Interactions*With	7.05	1.20–41.56	**0.031**	6.61	1.30–33.58	**0.023**
Approach*Without	15.31	1.88–124.72	**0.011**	1.46	0.17–12.49	0.732
Approach*With	5.54	0.96–31.91	0.055	3.32	0.35–31.39	0.296
Attack*Without	3.21	0.57–18.16	0.187	1.11	0.14–8.72	0.920
Attack*With	3.06	0.53–17.77	0.214	2.17	0.23–20.70	0.502
Interactions*Approach*Without	0.06	0.01–0.62	**0.018**	0.36	0.04–3.35	0.368
Interactions*Approach*With	0.06	0.01–0.68	**0.023**	0.22	0.02–2.48	0.220
Interactions*Attack*Without	0.24	0.03–2.13	0.202	0.50	0.06–4.06	0.515
Interactions*Attack*With	0.22	0.02–2.44	0.219	0.37	0.04–3.19	0.362


***Random Effects***		
*σ*^2^	3.29	3.29
*τ*_00sex_	2.18	0.43
*τ*_00color_	1.14	4.83
ICC	0.50	0.62
Marginal/Conditional R^2^	0.248/0.626	0.020/0.623

**Notes.**

CI 0.95 confidence interval *σ*^2^ within group variation *τ*_00_ between group variation on intercept ICC interclass coefficient (proportion explained by the grouping structure)

Values in bold refer to *p* < 0.05.

**Figure 5:**
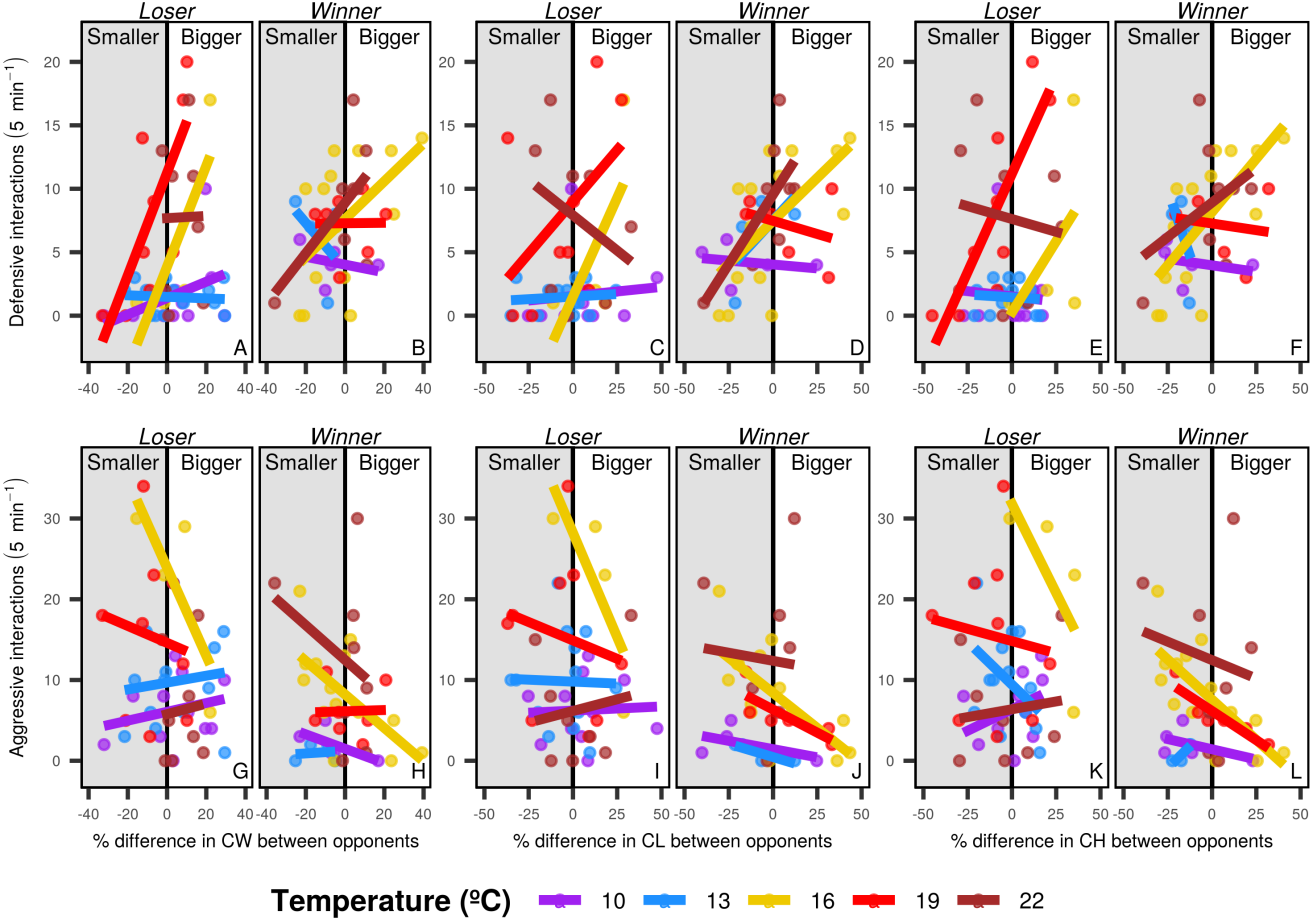
Defensive and aggressive interactions of shore crabs. Relationship between the number of defensive and aggressive interactions and the differences in size of different morphological structures of shore crabs (*Carcinus maenas*) involved in resource disputes. Only one individual had their behavior recorded during the dispute, with the crab holding the food after the 5 minute period being considered the winner of the dispute. CW, carapace width, CL, chela length and CH, chela height. Defensive (A to F) and aggressive interactions (G to L) and losers (A, C, E, G, I and K) and winners (B, D, F, H, J and L) are shown in separated sub-panels.

Overall, individuals with bigger body and chela sizes displayed more defensive interactions than smaller individuals, whereas smaller crabs had more aggressive interactions. The pattern differed among temperature levels, with marginal effects in cooler temperatures (below 10 and 13 °C) and higher intensity at the intermediate temperature level (16 °C). In warm waters (19 and 22 °C) the morphological dissimilarities between contestants were not obvious, but the smaller loser crabs displayed more aggressive pattern than in the other temperature levels (especially regarding chela morphology) (Fig. 5).

## Discussion

The findings of the study suggest that the agonistic behavior of the shore crab is influenced by the interaction of morphological, physiological and environmental conditions present at a given dyadic contest. In general, the differences in carapace width between opponents, their color morphotype, sex and temperature conditions interacted and were important predictors of the contest fate.

Water temperature influenced the frequency of agonistic events, with crabs being more agitated in warmer temperatures as expected for an ectothermic animal (Hoffman, Dunham & Kelly, 1975). According to Thorp (1978), agonistic interactions increase with temperature in ectotherms and start to decline as the thermal limits are reached. At temperatures above 16 °C agonistic interactions were observed more frequently, whereas at 10 °C crabs were mostly inactive, displaying fewer agonistic behaviors than individuals in warmer conditions. The shore crab is a highly tolerant species to environmental constraints, being able to survive in temperatures varying from 0 to 30 °C (Cohen, Carlton & Fountain, 1995). However, regardless of their broad tolerance, *C. maenas* seem to have their feeding impetus inhibited at 7 °C (Ropes, 1968), while increasing their feeding rates at higher temperatures (c.a. 17 °C) (Wallace, 1973), which is similar to the observed thermal peak of aggressiveness in our study (16 °C). Even though the temperature range investigated in our study did not encompass the entire spectrum of temperatures in which the shore crab can live, it approached the lower limit where appetite is inhibited (Ropes, 1968). The upper limit of our observations was nearly 4 °C higher than commonly found in the warmest season in the geographical region where the crabs were collected (Baeta et al., 2005; Souza, 2013). Portugal is near the edge of the southern limit of the native distribution of the shore crab (Roman & Palumbi, 2004), and due to the history of failed invasions of *C. maenas* in warmer waters (see Klassen & Locke, 2007), and the sluggish behavior that some individuals demonstrated at 22 °C, we do believe that the upper temperature limit set in our study was reasonable to test the effects of warming waters without putting the observed crabs into unnecessary and extreme thermal stress.

The risk of carapace damage and the costs associated with it is greater in colder waters (Juanes & Hartwick, 1990), due to the higher solubility of calcium carbonate (Vermeij, 1995; Juanes & Hartwick, 1990) and to the slower molting rates (Audet et al., 2003) at lower temperatures. In addition, the carapace hardening time is up to four times faster in warmer than in colder waters (Crothers, 1967). These conditions can lead to long recovery times for damaged crabs in cold waters, which might be an incentive to be more cautious during contests, and also explains the less aggressive behavior of *C. maenas* at lower temperatures observed in this study. High temperatures, on the other hand, accelerate the physiological processes in ectothermic animals, including enzymatic reactions that regulate their metabolism. As such, faster digestion is expected to occur in higher temperatures, contributing to higher demands for food. Hence, animals are expected to increase their foraging activities, which consequently explains the higher rate of aggressive agonistic interactions observed at higher temperatures (Hoffman, Dunham & Kelly, 1975).

Overall, most of the individuals, when not holding the food, displayed more attacks during the before and the without context. This result suggests that an early use of aggressive behaviors in food contests might determine the contest outcome, increasing the chances that the crab would keep the resource (Huber et al., 1997). On the other hand, a more aggressive strategy could increase the risk of chela damage and limb loss. Injury in this species usually is followed by regeneration, but imposes an extra energetic demand (regenerative load), which reduces the energy allocation for somatic growth and/or reproductive investment (Mariappan, Balasundaram & Schmitz, 2000). The risk of physical injuries might also be related to the strategy adopted by some individuals that performed retreats even when not in possession of the food, indicating that a safer strategy can be advantageous in cases where their assessment of the opponent’s RHP suggests that they have minor chances to win the dispute. Interestingly, the individuals that won the food disputes displayed a higher rate of retreats, indicating that the crabs preferred to avoid physical confrontation than to actively defend the resource. This strategy was also observed in previous laboratory studies, in which the dominant individuals opted to resolve most of the outcomes through the use of retreats and opponent avoidance (Karavanich & Atema, 1998; Spanier et al., 1998).

Body size was a very a relevant predictor of the contest outcomes, with most of the dyadic contests being won by larger individuals. In fact, larger body size often translates to greater strength in crabs (Preston et al., 1996), which increases the individual foraging success and its RHP (Lee, 1993; Sneddon, Huntingford & Taylor, 1997b). Our results showed that a larger body size was important in determining the contest outcomes in cold water conditions, but not in warm waters. In warm waters the contest outcomes seemed to have a higher level of unpredictability, given that the advantages of bigger body sizes might have been lost due to physiological constraints on larger crabs. This result can be explained by differential oxygen limitation thresholds experienced by small and large aquatic ectotherms in warm conditions, being harsher for the latter (Bell, Davidson & Scarborough, 1968; Hart, 1980). Therefore, smaller crabs might have been less limited by oxygen than bigger ones in the contests that took place at warm conditions, corroborating previous findings (Marsden, Newell & Ahsanullah, 1973). High water temperature can also affect the hemolymph’s oxygen carrying capacity, which worsens individual reactions to hypoxia, and increases its vulnerability to other stressors (Camus et al., 2004; Dejours, Toulmond & Truchot, 1985). Consequently, in scenarios of increased temperatures, larger crabs lose competitiveness to smaller individuals. This pattern can contribute to the intensification of the ontogenetic habitat partitioning observed in this species, putting an additional energetic load on individuals that probably will have to engage in longer migration routes, altering the mortality pressure experienced in the population (Klaassen et al., 2014; Sillett & Holmes, 2002).

In *C. maenas* the color morphotype indicates different physiological conditions related to the molt status of the individuals, with the color red being more commonly observed in water with higher salinity and older (larger) individuals, and characterized by individuals with thicker exoskeletons (Sneddon et al., 2000; Baeta et al., 2005). On the other hand, green crabs sometimes comprise recently molted individuals that are usually characterized as having a thinner exoskeleton (Sneddon et al., 2000), but this is not always the case (Souza et al., 2011). All individuals used in the experiment came from the same habitat at an estuarine zone (c.a. 1.5 km from the river mouth) where it is common to find both color morphotypes, with the green morphotype being more frequently observed (Souza, 2013). In the present study, color morphotype was an important predictor of the contest outcomes, suggesting that the physiological mechanisms associated with it played an important role in the observed agonistic interactions, regardless of the temperature effect. In asymmetrical disputes between red and green crabs, the former won most of the contests for food at lower temperature levels (10 to 16 °C), but the opposite pattern was observed in warmer waters. Usually, green and red crabs have different habitat preferences with red crabs inhabiting deeper and colder waters compared to green crabs (Baeta et al., 2005; Souza, 2013), reflecting the different responses of individual crabs to cope with environmental conditions (Abelló et al., 1997; McGaw & Naylor, 1992; Warman, Reid & Naylor, 1993). The shift in dominance of a given color morphotype across different temperature levels indicates that the thermal optimal level must be different between color morphotypes, and that the red morphotype loses its dominance when the water temperature crosses the threshold of 19 °C. It is important to note also that red crabs are sexually mature and are usually larger than the green ones, so the observed effects must be regarded with caution due to the complex levels of interactions among the different morphological and physiological traits observed in this species. Nevertheless, our results indicate that different morphotypes thrive at different temperature ranges and this can be one of the drivers of the spatial segregation of *C. maenas* populations.

The sex of the individuals is also a relevant morphological attribute in the agonistic contests. Overall, most of the studies performed with animal contests have focused in contest between members of the same sex, with few studies looking at inter-sexual contests (but see Briffa & Dallaway, 2007). Inter-sexual crustacean dyadic contests are controversial, with some researchers reporting relevant effects of the individual’s sex in the number of agonistic interactions during the resource disputes (Villanelli & Gherardi, 1998; Warren et al., 2009), while others reporting marginal effects (Briffa & Dallaway, 2007). Briffa & Dallaway (2007) observed that males and females of the species *Pagurus bernhardus* have great differences in their agonistic behavior and suggested that overall, males seem to have an advantage compared to females when attacking and defending. In the present study, no clear pattern was observed regarding the sex of the individuals and the contests outcomes, and this result corroborates with previous studies made with the hermit crabs *Pagurus longicarpus* and *Calcinus tibicen* (Hazlett, 1966; Winston & Jacobson, 1978). From our results it is possible to conclude that sex is an important predictor of the fate of food disputes, but also might not be as important as size and color morphotype, corroborating with other studies on crustaceans (Briffa & Dallaway, 2007).

Given the invasion history of *C. maenas* in several ecosystems worldwide (Roman & Palumbi, 2004) and the increased aggressiveness of the species in higher temperatures (this study), it is expected that the effects of this invasive aquatic species (IAS) will be further aggravated in some systems where temperatures are currently low (lower than the 19 °C). On the other hand, in areas where the population already lives in high temperatures, a further increase in temperature might have detrimental physiological and behavioral consequences for the shore crab population, leading to a stronger spatial segregation in the populations or a decline in their densities.

Most studies on animal contests focus on symmetrical disputes, but in nature, symmetrical disputes might not be as frequent as asymmetrical ones. Even though there has been some effort to understand asymmetrical disputes in previous years (e.g., Briffa & Dallaway, 2007; Couterne-Jones & Briffa, 2014; Kotiaho et al., 1999; Wiltenmuth, 1996), the topic is far from fully explored by ethologists. Due to complex interactions among the different morphological and physiological conditions of *C. maenas* in the environment, understanding the roles played by each variable is a very hard task to accomplish. Nevertheless, our results shed light on the topic, demonstrating that morphological, physiological and environmental factors interact to create a complex puzzle of behaviors that is not easily disentangled.

## Conclusions

In summary, we found that shore crab morphology and color morphotype are determinants in the fate of dyadic disputes, but different temperatures create dissimilar behavioral responses, with higher temperatures disrupting the well-established dominance of bigger individuals of the red color morphotype, suggesting that *C. maenas* agonistic contests might have a higher plasticity than previously acknowledged.

##  Supplemental Information

10.7717/peerj.7845/supp-1 Data S1 Raw data

## Additional Information and Declarations

### Field Study Permissions

The following information was supplied relating to field study approvals (i.e., approving body and any reference numbers): Permission to collect shore crabs in the Minho estuary (NW Iberian Peninsula) was granted in correspondence with a captain of the Portuguese Navy.

### Data Availability

The following information was supplied regarding data availability: The raw data are available as a Supplemental File.
